# Crystal structures of 2-methyl­pyridinium hydrogen 2,3-bis­(4-methyl­benzo­yloxy)succinate and bis-[4-methyl­pyridinium hydrogen 2,3-bis­(4-methyl­benzo­yloxy)succinate] penta­hydrate

**DOI:** 10.1107/S2056989017012981

**Published:** 2017-09-15

**Authors:** P. Sivakumar, S. Israel, G. Chakkaravarthi

**Affiliations:** a Research and Development, Centre, Bharathiar University, Coimbatore 641 046, India; b Department of Physics, CPCL Polytechnic College, Chennai 600 068, India; c Post Graduate and Research Department of Physics, The American College, Madurai-625 002, India

**Keywords:** succinates, pyridinium salts, hydrogen bonding, crystal structure

## Abstract

The title salt (I), C_6_H_8_N^+^·C_20_H_17_O_8_^−^, comprises a 2-methyl­pyridinium cation and a 2,3-bis­(4-methyl­benzo­yloxy)succinate mono-anion while the salt (II), 2C_6_H_8_N^+^·2C_20_H_17_O_8_^−^·5H_2_O, consists of a pair of 4-methyl­pyridinium cations and 2,3-bis­(4-methyl­benzo­yloxy)succinate mono-anions and five water mol­ecules of solvation in the asymmetric unit. In (I), the dihedral angle between the aromatic rings of the anion is 40.41 (15)°, comparing with 43.0 (3) and 85.7 (2)° in the conformationally dissimilar anion mol­ecules in (II). The pyridine ring of the cation in (I) is inclined at 23.64 (16) and 42.69 (17)° to the two benzene moieties of the anion. In (II), these comparative values are 4.7 (3), 43.5 (3)° and 43.5 (3), 73.1 (3)° for the two associated cation and anion pairs. The crystal packing of (I) is stabilized by inter-ionic N—H⋯O, O—H⋯O and C—H⋯O hydrogen bonds as well as weak C—H⋯π inter­actions, linking the ions into infinite chains along [100]. In the crystal packing of (II), the anions and cations are also linked by N—H⋯O and O—H⋯O hydrogen bonds involving also the water mol­ecules, giving a two-dimensional network across (001). The crystal structure is also stabilized by weak C—H⋯O and C—H⋯π inter­actions.

## Abstract 2

The 1:1, 2-methyl­pyridium and 4-methyl­pyridinium salts of the chiral 4-methyl­benzo­yloxy-substituted succinic acid form, respectively one- and two-dimensional hydrogen-bonded crystal structures,

## Chemical context   

Pyridine derivatives exhibit biological activities such as anti­viral (Hamdouchi *et al.*, 1999 ▸), anti­bacterial (Rival *et al.*, 1992 ▸), anti­microbial (Jo *et al.*, 2004 ▸), anti­thrombotic (Sunkel *et al.*, 1990 ▸). Some pyridine derivatives possess non-linear optical (NLO) properties (Tomaru *et al.*, 1991 ▸) and often possess anti­bacterial and anti­fungal activities (Akkurt *et al.*, 2005 ▸). We have now synthesized and determined the crystal structures of the title 1:1 salts of the chiral diprotic acid, 2,3-bis­(4-methyl­benzo­yloxy)succinic acid with 2-methyl­pyridine, C_6_H_8_N^+^·C_20_H_17_O_8_^−^, (I), and with 4-methyl­pyridine, 2C_6_H_8_N^+^·2C_20_H_17_O_8_^−^·5H_2_O, (II).

## Structural commentary   

**Figure 1:**
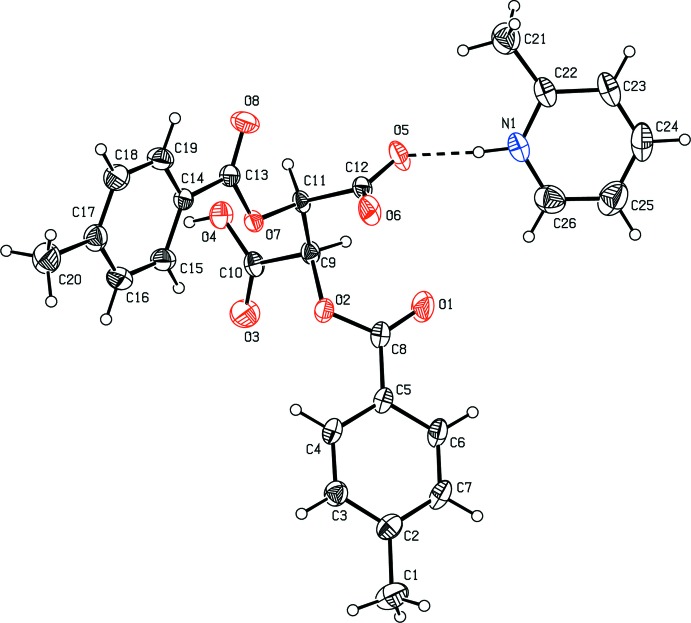
The mol­ecular structure and atom numbering scheme in the title salt (I), with 30% probability displacement ellipsoids. The inter-species hydrogen bond is shown as a dashed line.

**Figure 2:**
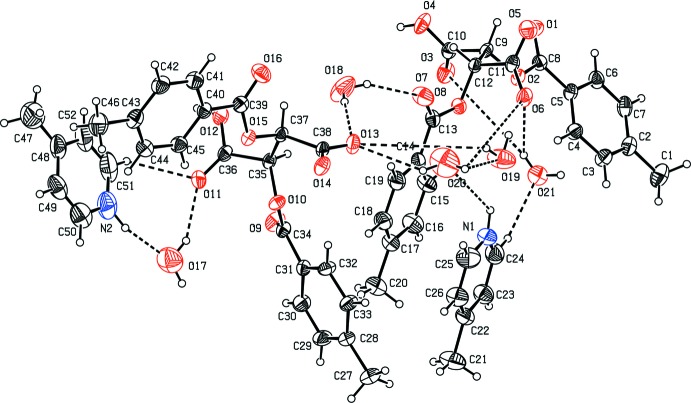
The mol­ecular structure of the two independent cation and anion pairs and the water mol­ecules of solvation in the asymmetric unit of the title salt (II), with 30% probability displacement ellipsoids. Inter-species hydrogen bonds are shown as dashed lines.

In both the salts of 2,3-bis­(4-methyl­benzo­yloxy)succinic acid [(I) and (II), Figs. 1 ▸ and 2 ▸, respectively], the N atoms of the pyridine mol­ecules are protonated. With (I), the asymmetric unit comprises a single 2-methyl­pyridinium cation and a succinate mono-anion (Fig. 1 ▸) whereas with (II), the asymmetric unit comprises two 4-methyl­pyridinium cations and two succinate mono-anions along with five water mol­ecules of solvation (Fig. 2 ▸). In salt (I), the dihedral angle between the aromatic rings (C2–C7) and (C14–C19) is 40.41 (15)°. The pyridine ring (N1/C22–C26) is inclined at angles of 23.64 (16) and 42.69 (17)° with the benzene rings (C2–C7) and (C14–C19), respectively. In salt (II), the benzene ring (C2–C7) forms a dihedral angle of 43.0 (3)° with the benzene ring (C14–C19) whereas the benzene ring (C40–C45) and (C28–C33) are inclined at an angle of 85.7 (2)°. The dihedral angles between the pyridine ring (C22/C23/C24/N1/C25/C26) and the benzene rings (C2–C7) and (C14–C19) are 43.5 (3) and 4.7 (3)°, respectively, and those between the pyridine ring (C48/C49/C50/N2/C51/C52) and the benzene rings (C28–C33) and (C40–C45) are 73.1 (3) and 43.5 (3)°, respectively.
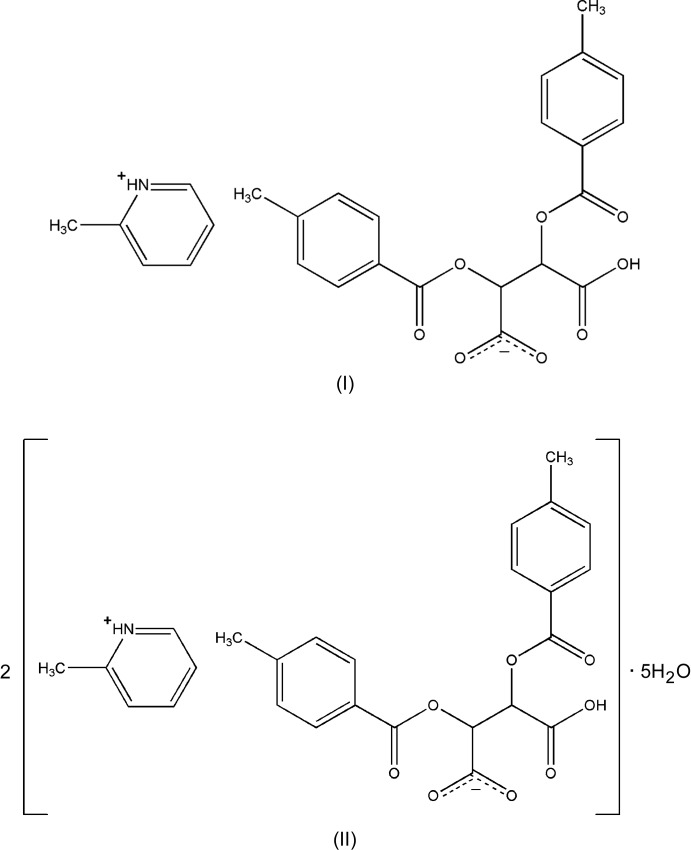


## Supra­molecular features   

**Figure 3:**
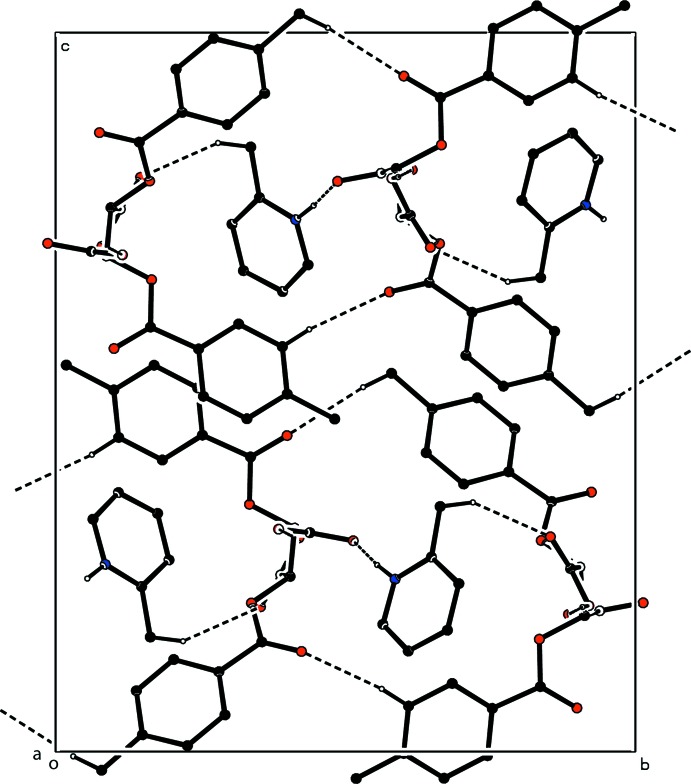
The crystal packing of the title salt (I) in the unit cell, viewed along the *a* axis. The hydrogen bonds are shown as dashed lines and H atoms not involved in hydrogen bonding have been omitted.

**Figure 4:**
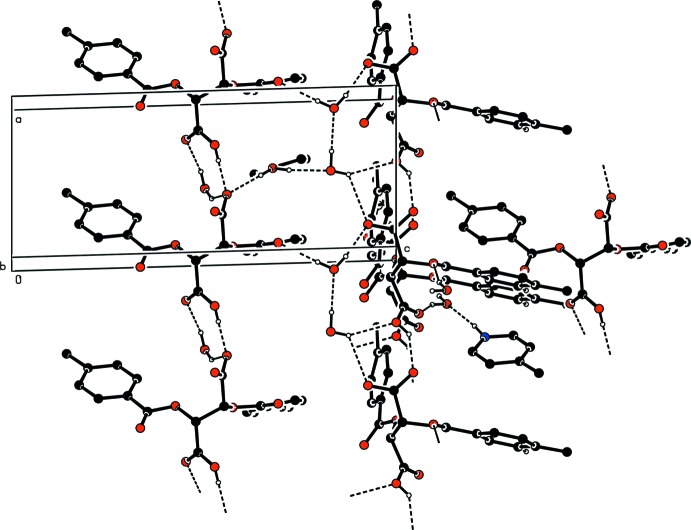
The crystal packing of the title salt (II) in the unit cell, viewed along the *b* axis. The hydrogen bonds are shown as dashed lines. H atoms not involved in hydrogen bonding have been omitted.

The crystal structure of (I) is stabilized by intra-ionic N—H⋯O, inter-ionic O—H⋯O, C—H⋯O (Table 1 ▸, Fig. 3 ▸) and C—H⋯π (Table 1 ▸) inter­actions. The inter-ionic O—H⋯O hydrogen bond links the ions into an infinite chain along [100]. In the crystal packing of (II), the cations and anions are linked by N—H⋯O and O—H⋯O hydrogen bonds (Table 2 ▸, Fig. 4 ▸), through water mol­ecules, giving an infinite two-dimensional network parallel to (001). The structure is further influenced by weak C—H⋯O hydrogen-bonding inter­actions and weak C—H⋯π contacts (Table 2 ▸) while there are also very weak π–π inter­actions between like pyridine rings [minimum ring-centroid separations *Cg*1⋯*Cg*6^i^, 3.996 (4) Å and *Cg*2⋯*Cg*5^ii^, 3.900 (3) Å where *Cg*1, *Cg*2, *Cg*5 and *Cg*6 are the centroids of the C2–C7, C14–C19, N1/C22–C26 and N2/C48–C52 rings, respectively; symmetry codes: (i) 1 + *x*, 1 + *y*, −1 + *z*; (ii) *x*, *y*, *z*].

## Database survey   

The geometric parameters of the cation of (I), which contains 2-methyl pyridinium, are comparable with the reported crystal sructures of 2-methyl­pyridinium 2-carb­oxy­benzoate-benzene-1,2-di­carb­oxy­lic acid (2/1) (Sivakumar, Sudhahar, Gunasekaran *et al.*, 2016 ▸); 2-methyl­pyridinium 2-carb­oxy-6-nitro­benzoate (Sivakumar, Sudhahar Israel *et al.*, 2016 ▸); 2-methyl­pyridinium 5-(2,4-di­nitro­phen­yl)-1,3-di­methyl­barbiturate (Sridevi & Kalaivani, 2012 ▸). The geometric parameters of the 4-methyl­pyridinium cation of (II) are comparable with those reported in the crystal structures of 4-methyl­pyridinium 2-carb­oxy-6-nitro­benzoate (Devi *et al.*, 2016 ▸), 4-methyl­pyridinium 4-hy­droxy­benzoate (Sudhahar *et al.*, 2013 ▸) and 4-methyl­pyridinium 2-carb­oxy-4,5-di­chloro­benzoate monohydrate (Smith & Wermuth, 2010 ▸). The geometric parameters of anions of (I) and (II) are comparable with the reported structures of 2,3-di-*p*-tolyl-(2*R*,3*R*)-tartaric acid ethyl acetate solvate (Tang *et al.*, 2006 ▸) and di-*p*-tolyl­tartaric acid with aromatic amines (Nassimbeni & Su, 2006 ▸).

## Synthesis and crystallization   

The title salts (I) and (II) were synthesized using the reaction of equi-molar qu­anti­ties of di-*p*-tolyl-l-tartaric acid (0.967 g) and 0.237 g of either 2-methyl­pyridine [for (I)] or 4-methyl­pyridine [for (II)], dissolved in 10 ml of acetone. A white precipitate was formed, which was dissolved in 30 ml of water and then kept at room temperature for slow evaporation. After 2 months, crystals of (I) or (II), suitable for X-ray diffraction analysis were obtained.

## Refinement   

Crystal data, data collection and structure refinement details are summarized in Table 3 ▸. C-bound H atoms were placed in calculated positions and allowed to ride on their carrier atoms, with C—H = 0.93 Å (aromatic CH), 0.98 Å for CH, or 0.96 Å (methyl CH), and with *U*_iso_ = 1.5*U*_eq_(methyl C or O) and *U*_iso_ = 1.2*U*_eq_(aromatic and methyl­ene C). H atoms for NH and OH groups were located in difference-Fourier maps and refined with a distance restraint [N—H = 0.86 (1) Å or O—H = 0.82 (1) Å]. The Flack absolute structure obtained for both structures (Parsons *et al.*, 2013 ▸) for the arbitrarily numbered chiral atoms [C9*R*,C11*R*] gave ambiguous Flack parameters of 0.4 (4) [(for (I)] and 0.6 (3) [for (II)], for 1335 and 2690 quotients, respectively.

## Supplementary Material

Crystal structure: contains datablock(s) I, II, global. DOI: 10.1107/S2056989017012981/zs2388sup1.cif

Structure factors: contains datablock(s) I. DOI: 10.1107/S2056989017012981/zs2388Isup2.hkl

Structure factors: contains datablock(s) II. DOI: 10.1107/S2056989017012981/zs2388IIsup3.hkl

Supporting information file. DOI: 10.1107/S2056989017012981/zs2388Isup4.cml

CCDC references: 1573939, 1573938

Additional supporting information:  crystallographic information; 3D view; checkCIF report

## Figures and Tables

**Table 1:** Hydrogen-bond geometry (Å, °) for (I) *Cg*1 is the centroid of the C2–C7 ring.

*D*—H⋯*A*	*D*—H	H⋯*A*	*D*⋯*A*	*D*—H⋯*A*
N1—H1⋯O5	0.87 (1)	1.74 (2)	2.593 (3)	166 (3)
O4—H4*A*⋯O6^i^	0.84 (1)	1.67 (1)	2.509 (2)	175 (5)
C1—H1*A*⋯O8^ii^	0.96	2.58	3.522 (4)	168
C16—H16⋯O1^ii^	0.93	2.51	3.362 (4)	153
C21—H21*A*⋯O3^iii^	0.96	2.38	3.238 (4)	148
C7—H7⋯*Cg*1^iv^	0.93	2.89	3.5882 (1)	133
C21—H21*B*⋯*Cg*1	0.96	2.91	3.7651 (1)	148

Symmetry codes: (i) 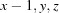; (ii) 
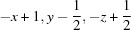
; (iii) 
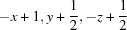
; (iv) 
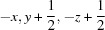
.

**Table 2:** Hydrogen-bond geometry (Å, °) for (II) *Cg*1 and *Cg*4 are the centroids of the C2–C7 and C40–C45 rings, respectively.

*D*—H⋯*A*	*D*—H	H⋯*A*	*D*⋯*A*	*D*—H⋯*A*
C24—H24⋯O21	0.93	2.41	3.262 (8)	152
C51—H51⋯O11	0.93	2.43	3.072 (10)	127
O4—H4*A*⋯O6^i^	0.82	1.69	2.503 (4)	170
O12—H12⋯O14^i^	0.82	1.80	2.472 (4)	138
N2—H2⋯O17	0.97 (3)	1.89 (3)	2.832 (10)	164 (5)
O17—H17*A*⋯O16^ii^	0.88 (3)	2.48 (3)	3.330 (7)	162 (7)
O17—H17*B*⋯O11	0.89 (3)	2.03 (4)	2.828 (7)	148 (7)
O18—H18*B*⋯O7	0.85 (3)	2.09 (5)	2.894 (6)	157 (9)
O18—H18*A*⋯O13	0.84 (3)	2.00 (4)	2.815 (6)	164 (9)
O19—H19*A*⋯O18^iii^	0.91 (3)	2.22 (5)	3.090 (11)	160 (11)
O19—H19*B*⋯O12^iii^	0.88 (3)	2.32 (5)	3.115 (7)	151 (7)
O19—H19*B*⋯O13	0.88 (3)	2.52 (6)	3.048 (8)	120 (6)
O20—H20*E*⋯O6	0.91 (3)	2.00 (3)	2.867 (6)	159 (6)
O20—H20*D*⋯O19	0.88 (3)	1.97 (4)	2.717 (7)	142 (6)
O21—H21*E*⋯O3^iii^	0.85 (3)	2.19 (4)	2.976 (5)	154 (6)
O21—H21*D*⋯O6	0.82 (3)	2.25 (5)	2.914 (5)	139 (5)
N1—H1⋯O20	0.88 (3)	1.77 (3)	2.644 (6)	172 (6)
C41—H41⋯*Cg*1^iv^	0.93	2.90	3.468 (6)	121
C47—H47*A*⋯*Cg*4^i^	0.96	2.94	3.707 (10)	137

Symmetry codes: (i) 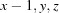; (ii) 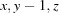; (iii) 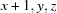; (iv) 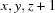.

**Table 3:** Experimental details

	(I)	(II)
Crystal data		
Chemical formula	C_20_H_17_O_8_^+^·C_6_H_8_N^−^	2C_20_H_17_O_8_^+^·2C_6_H_8_N^−^·5H_2_O
*M*_r_	479.47	1049.02
Crystal system, space group	Orthorhombic, *P*2_1_2_1_2_1_	Triclinic, *P*1
Temperature (K)	296	296
*a*, *b*, *c* (Å)	7.4849 (2), 16.2063 (4), 20.0959 (7)	7.5106 (2), 10.0155 (3), 18.5203 (5)
α, β, γ (°)	90, 90, 90	75.646 (2), 88.438 (2), 86.344 (2)
*V* (Å^3^)	2437.68 (12)	1346.81 (7)
*Z*	4	1
Radiation type	Mo *K*α	Mo *K*α
μ (mm^−1^)	0.10	0.10
Crystal size (mm)	0.30 × 0.26 × 0.24	0.40 × 0.30 × 0.30

Data collection		
Diffractometer	Bruker APEXII CCD Diffractometer	Bruker APEXII CCD Diffractometer
Absorption correction	Multi-scan (*SADABS*; Bruker, 2004 ▸)	Multi-scan (*SADABS*; Bruker, 2004 ▸)
*T*_min_, *T*_max_	0.707, 0.746	0.683, 0.746
No. of measured, independent and observed [*I* > 2σ(*I*)] reflections	23766, 7045, 4206	26169, 9433, 6749
*R*_int_	0.038	0.031
(sin θ/λ)_max_ (Å^−1^)	0.709	0.595

Refinement		
*R*[*F* ^2^ > 2σ(*F* ^2^)], *wR*(*F* ^2^), *S*	0.053, 0.124, 1.01	0.044, 0.121, 1.02
No. of reflections	7045	9433
No. of parameters	326	715
No. of restraints	2	21
H-atom treatment	H atoms treated by a mixture of independent and constrained refinement	H atoms treated by a mixture of independent and constrained refinement
Δρ_max_, Δρ_min_ (e Å^−3^)	0.20, −0.22	0.37, −0.23
Absolute structure	Flack *x* determined using 1335 quotients [(*I* ^+^)−(*I* ^−^)]/[(*I* ^+^)+(*I* ^−^)] (Parsons *et al.*, 2013 ▸).	Flack *x* determined using 2690 quotients [(*I* ^+^)−(*I* ^−^)]/[(*I* ^+^)+(*I* ^−^)] (Parsons *et al.*, 2013 ▸)
Absolute structure parameter	0.4 (4)	0.6 (3)

Computer programs: *APEX2* and *SAINT* (Bruker, 2004 ▸), *SHELXS2016* (Sheldrick, 2008 ▸), *SHELXL2016* (Sheldrick, 2015 ▸) and *PLATON* (Spek, 2009 ▸).

## supplementary crystallographic information

### 2-Methylpyridinium hydrogen 2,3-bis(4-methylbenzoyloxy)succinate (I) Crystal data

**Table:**

C_20_H_17_O_8_^+^·C_6_H_8_N^−^	*D*_x_ = 1.306 Mg m^−^^3^
*M**_r_* = 479.47	Mo *K*α radiation, λ = 0.71073 Å
Orthorhombic, *P*2_1_2_1_2_1_	Cell parameters from 5788 reflections
*a* = 7.4849 (2) Å	θ = 2.3–24.3°
*b* = 16.2063 (4) Å	µ = 0.10 mm^−^^1^
*c* = 20.0959 (7) Å	*T* = 296 K
*V* = 2437.68 (12) Å^3^	Block, colourless
*Z* = 4	0.30 × 0.26 × 0.24 mm
*F*(000) = 1008	

### 2-Methylpyridinium hydrogen 2,3-bis(4-methylbenzoyloxy)succinate (I) Data collection

**Table:**

Bruker APEXII CCD Diffractometer	4206 reflections with *I* > 2σ(*I*)
ω and φ scans	*R*_int_ = 0.038
Absorption correction: multi-scan (SADABS; Bruker, 2004)	θ_max_ = 30.3°, θ_min_ = 2.4°
*T*_min_ = 0.707, *T*_max_ = 0.746	*h* = −10→10
23766 measured reflections	*k* = −21→22
7045 independent reflections	*l* = −27→28

### 2-Methylpyridinium hydrogen 2,3-bis(4-methylbenzoyloxy)succinate (I) Refinement

**Table:**

Refinement on *F*^2^	Hydrogen site location: mixed
Least-squares matrix: full	H atoms treated by a mixture of independent and constrained refinement
*R*[*F*^2^ > 2σ(*F*^2^)] = 0.053	*w* = 1/[σ^2^(*F*_o_^2^) + (0.0586*P*)^2^ + 0.0095*P*] where *P* = (*F*_o_^2^ + 2*F*_c_^2^)/3
*wR*(*F*^2^) = 0.124	(Δ/σ)_max_ < 0.001
*S* = 1.01	Δρ_max_ = 0.20 e Å^−^^3^
7045 reflections	Δρ_min_ = −0.22 e Å^−^^3^
326 parameters	Absolute structure: Flack *x* determined using 1335 quotients [(*I*^+^)-(*I*^-^)]/[(*I*^+^)+(*I*^-^)] (Parsons *et al.*, 2013).
2 restraints	Absolute structure parameter: 0.4 (4)

### 2-Methylpyridinium hydrogen 2,3-bis(4-methylbenzoyloxy)succinate (I) Special details

**Table:**

Geometry. All esds (except the esd in the dihedral angle between two l.s. planes) are estimated using the full covariance matrix. The cell esds are taken into account individually in the estimation of esds in distances, angles and torsion angles; correlations between esds in cell parameters are only used when they are defined by crystal symmetry. An approximate (isotropic) treatment of cell esds is used for estimating esds involving l.s. planes.

### 2-Methylpyridinium hydrogen 2,3-bis(4-methylbenzoyloxy)succinate (I) Fractional atomic coordinates and isotropic or equivalent isotropic displacement parameters (Å^2^)

**Table:**

	*x*	*y*	*z*	*U*_iso_*/*U*_eq_	
C2	0.6147 (4)	0.14980 (19)	0.02329 (15)	0.0504 (7)	
C3	0.5336 (4)	0.13935 (17)	0.08445 (15)	0.0486 (7)	
H3	0.492129	0.087332	0.096414	0.058*	
C4	0.5128 (4)	0.20389 (16)	0.12799 (15)	0.0429 (7)	
H4	0.457560	0.195386	0.168872	0.051*	
C5	0.5740 (4)	0.28162 (15)	0.11098 (14)	0.0395 (6)	
C6	0.6610 (4)	0.29218 (18)	0.05099 (16)	0.0512 (8)	
H6	0.707142	0.343624	0.039763	0.061*	
C7	0.6797 (4)	0.22748 (19)	0.00812 (17)	0.0571 (9)	
H7	0.737457	0.235793	−0.032292	0.069*	
C1	0.6274 (6)	0.0795 (2)	−0.02603 (19)	0.0760 (11)	
H1A	0.577360	0.030495	−0.006707	0.114*	
H1B	0.750508	0.069861	−0.037016	0.114*	
H1C	0.562519	0.093486	−0.065651	0.114*	
C8	0.5389 (4)	0.35568 (16)	0.15234 (15)	0.0435 (7)	
C9	0.3662 (3)	0.40446 (15)	0.24351 (14)	0.0359 (6)	
H9	0.386796	0.455832	0.218959	0.043*	
C10	0.1651 (3)	0.38826 (16)	0.24566 (16)	0.0413 (7)	
C11	0.4517 (3)	0.41223 (13)	0.31095 (14)	0.0328 (6)	
H11	0.387318	0.454192	0.336611	0.039*	
C12	0.6476 (3)	0.43854 (15)	0.30436 (14)	0.0384 (6)	
C13	0.4645 (3)	0.33626 (15)	0.40973 (14)	0.0378 (6)	
C14	0.4553 (3)	0.25329 (14)	0.44002 (14)	0.0363 (6)	
C15	0.4030 (4)	0.18421 (17)	0.40542 (16)	0.0494 (7)	
H15	0.372208	0.188437	0.360708	0.059*	
C16	0.3961 (5)	0.10887 (17)	0.43680 (17)	0.0558 (9)	
H16	0.360245	0.062617	0.412886	0.067*	
C17	0.4412 (4)	0.10048 (16)	0.50286 (17)	0.0519 (8)	
C18	0.4951 (5)	0.17001 (18)	0.53717 (16)	0.0567 (8)	
H18	0.525612	0.165861	0.581900	0.068*	
C19	0.5040 (5)	0.24497 (18)	0.50593 (17)	0.0504 (7)	
H19	0.543369	0.290919	0.529441	0.060*	
C20	0.4279 (6)	0.01852 (18)	0.53753 (19)	0.0741 (11)	
H20A	0.516937	0.015397	0.571817	0.111*	
H20B	0.446815	−0.025036	0.505937	0.111*	
H20C	0.311381	0.012861	0.556994	0.111*	
C26	0.9766 (5)	0.56457 (19)	0.1769 (2)	0.0700 (10)	
H26	0.918452	0.519393	0.158339	0.084*	
C25	1.0947 (7)	0.6084 (2)	0.1398 (2)	0.0863 (13)	
H25	1.121029	0.593244	0.096261	0.104*	
C24	1.1741 (5)	0.6759 (2)	0.1688 (3)	0.0831 (13)	
H24	1.251831	0.708392	0.143993	0.100*	
C23	1.1402 (5)	0.6956 (2)	0.2335 (2)	0.0695 (11)	
H23	1.196223	0.740906	0.252866	0.083*	
C22	1.0225 (4)	0.64833 (17)	0.27049 (17)	0.0510 (8)	
C21	0.9800 (5)	0.6634 (2)	0.34083 (19)	0.0748 (11)	
H21A	0.944156	0.719825	0.346523	0.112*	
H21B	1.083626	0.652551	0.367586	0.112*	
H21C	0.884402	0.627622	0.354348	0.112*	
N1	0.9432 (3)	0.58574 (14)	0.23975 (15)	0.0499 (6)	
O1	0.5866 (4)	0.42423 (12)	0.13919 (13)	0.0834 (9)	
O2	0.4435 (2)	0.33775 (10)	0.20649 (9)	0.0408 (4)	
O3	0.0897 (3)	0.35352 (16)	0.20132 (14)	0.0797 (8)	
O4	0.0887 (2)	0.41986 (12)	0.29719 (12)	0.0515 (5)	
O5	0.6695 (3)	0.51344 (11)	0.29317 (12)	0.0581 (6)	
O6	0.7639 (2)	0.38471 (11)	0.30924 (12)	0.0538 (6)	
O7	0.4337 (2)	0.33449 (9)	0.34376 (9)	0.0355 (4)	
O8	0.4950 (3)	0.39923 (11)	0.43943 (11)	0.0577 (6)	
H1	0.864 (4)	0.5548 (17)	0.2594 (16)	0.071 (11)*	
H4A	−0.021 (2)	0.409 (2)	0.299 (2)	0.107*	

### 2-Methylpyridinium hydrogen 2,3-bis(4-methylbenzoyloxy)succinate (I) Atomic displacement parameters (Å^2^)

**Table:**

	*U*^11^	*U*^22^	*U*^33^	*U*^12^	*U*^13^	*U*^23^
C2	0.0479 (17)	0.0592 (17)	0.0441 (19)	0.0101 (14)	0.0032 (14)	0.0005 (14)
C3	0.0537 (18)	0.0465 (15)	0.0457 (19)	0.0005 (14)	0.0045 (14)	0.0050 (13)
C4	0.0412 (15)	0.0513 (15)	0.0363 (17)	0.0006 (12)	0.0077 (12)	0.0094 (12)
C5	0.0338 (14)	0.0470 (14)	0.0378 (16)	0.0035 (12)	0.0073 (12)	0.0090 (12)
C6	0.0514 (18)	0.0523 (17)	0.050 (2)	0.0021 (14)	0.0194 (16)	0.0137 (14)
C7	0.059 (2)	0.069 (2)	0.043 (2)	0.0081 (16)	0.0214 (16)	0.0100 (16)
C1	0.096 (3)	0.075 (2)	0.057 (2)	0.014 (2)	0.010 (2)	−0.0102 (18)
C8	0.0385 (15)	0.0482 (15)	0.0438 (18)	−0.0018 (13)	0.0085 (13)	0.0097 (13)
C9	0.0275 (12)	0.0334 (13)	0.0468 (17)	0.0004 (10)	0.0061 (11)	0.0017 (11)
C10	0.0291 (13)	0.0449 (15)	0.0497 (19)	−0.0054 (11)	0.0003 (13)	0.0040 (14)
C11	0.0215 (11)	0.0283 (11)	0.0485 (17)	−0.0016 (9)	0.0050 (11)	0.0008 (11)
C12	0.0226 (12)	0.0440 (15)	0.0488 (18)	−0.0063 (11)	0.0030 (12)	−0.0051 (12)
C13	0.0296 (13)	0.0415 (14)	0.0423 (17)	−0.0023 (11)	0.0030 (12)	−0.0042 (12)
C14	0.0302 (13)	0.0381 (13)	0.0404 (17)	−0.0025 (10)	0.0052 (12)	−0.0034 (11)
C15	0.061 (2)	0.0454 (15)	0.0419 (18)	−0.0050 (14)	−0.0013 (14)	−0.0038 (14)
C16	0.070 (2)	0.0403 (16)	0.057 (2)	−0.0068 (14)	0.0044 (17)	−0.0072 (15)
C17	0.0524 (17)	0.0456 (16)	0.058 (2)	0.0025 (14)	0.0096 (16)	0.0052 (14)
C18	0.069 (2)	0.0575 (18)	0.0432 (19)	−0.0056 (16)	−0.0026 (15)	0.0055 (15)
C19	0.0593 (19)	0.0465 (15)	0.0452 (19)	−0.0059 (13)	−0.0071 (15)	−0.0057 (13)
C20	0.094 (3)	0.0509 (18)	0.077 (3)	0.0023 (19)	0.016 (2)	0.0117 (17)
C26	0.085 (3)	0.0507 (18)	0.074 (3)	0.0018 (18)	−0.005 (2)	0.0013 (17)
C25	0.108 (4)	0.072 (2)	0.079 (3)	0.017 (2)	0.031 (3)	0.015 (2)
C24	0.065 (2)	0.079 (3)	0.105 (4)	−0.008 (2)	0.031 (2)	0.027 (3)
C23	0.0501 (19)	0.061 (2)	0.097 (3)	−0.0210 (16)	0.002 (2)	0.015 (2)
C22	0.0379 (15)	0.0450 (16)	0.070 (2)	−0.0063 (13)	−0.0037 (14)	0.0135 (15)
C21	0.081 (3)	0.078 (2)	0.065 (3)	−0.012 (2)	−0.002 (2)	0.010 (2)
N1	0.0405 (13)	0.0422 (14)	0.067 (2)	−0.0073 (11)	−0.0005 (13)	0.0134 (12)
O1	0.117 (2)	0.0464 (12)	0.087 (2)	−0.0150 (13)	0.0531 (17)	0.0060 (12)
O2	0.0408 (10)	0.0384 (9)	0.0432 (11)	−0.0002 (8)	0.0126 (9)	0.0042 (8)
O3	0.0473 (12)	0.1178 (19)	0.0741 (18)	−0.0229 (13)	−0.0025 (12)	−0.0307 (16)
O4	0.0229 (9)	0.0668 (12)	0.0649 (15)	−0.0003 (9)	0.0041 (10)	−0.0068 (11)
O5	0.0414 (11)	0.0413 (11)	0.0916 (18)	−0.0156 (9)	0.0093 (11)	0.0040 (10)
O6	0.0197 (8)	0.0532 (11)	0.0886 (17)	0.0014 (8)	0.0088 (10)	0.0003 (11)
O7	0.0316 (9)	0.0338 (9)	0.0412 (11)	−0.0055 (7)	0.0030 (8)	0.0000 (8)
O8	0.0778 (16)	0.0406 (10)	0.0546 (14)	−0.0097 (10)	−0.0055 (12)	−0.0098 (9)

### 2-Methylpyridinium hydrogen 2,3-bis(4-methylbenzoyloxy)succinate (I) Geometric parameters (Å, º)

**Table:**

C2—C3	1.381 (4)	C14—C15	1.375 (4)
C2—C7	1.383 (4)	C14—C19	1.380 (4)
C2—C1	1.513 (4)	C15—C16	1.375 (4)
C3—C4	1.373 (4)	C15—H15	0.9300
C3—H3	0.9300	C16—C17	1.376 (4)
C4—C5	1.383 (4)	C16—H16	0.9300
C4—H4	0.9300	C17—C18	1.381 (4)
C5—C6	1.381 (4)	C17—C20	1.503 (4)
C5—C8	1.484 (4)	C18—C19	1.369 (4)
C6—C7	1.364 (5)	C18—H18	0.9300
C6—H6	0.9300	C19—H19	0.9300
C7—H7	0.9300	C20—H20A	0.9600
C1—H1A	0.9600	C20—H20B	0.9600
C1—H1B	0.9600	C20—H20C	0.9600
C1—H1C	0.9600	C26—N1	1.332 (5)
C8—O1	1.196 (3)	C26—C25	1.357 (6)
C8—O2	1.333 (3)	C26—H26	0.9300
C9—O2	1.435 (3)	C25—C24	1.374 (6)
C9—C11	1.504 (4)	C25—H25	0.9300
C9—C10	1.528 (4)	C24—C23	1.364 (6)
C9—H9	0.9800	C24—H24	0.9300
C10—O3	1.196 (3)	C23—C22	1.384 (4)
C10—O4	1.289 (4)	C23—H23	0.9300
C11—O7	1.428 (3)	C22—N1	1.328 (4)
C11—C12	1.533 (3)	C22—C21	1.469 (5)
C11—H11	0.9800	C21—H21A	0.9600
C12—O6	1.237 (3)	C21—H21B	0.9600
C12—O5	1.245 (3)	C21—H21C	0.9600
C13—O8	1.204 (3)	N1—H1	0.873 (13)
C13—O7	1.346 (3)	O4—H4A	0.839 (13)
C13—C14	1.478 (4)		
			
C3—C2—C7	117.5 (3)	C19—C14—C13	118.2 (2)
C3—C2—C1	121.2 (3)	C14—C15—C16	120.1 (3)
C7—C2—C1	121.3 (3)	C14—C15—H15	119.9
C4—C3—C2	121.6 (3)	C16—C15—H15	119.9
C4—C3—H3	119.2	C15—C16—C17	121.4 (3)
C2—C3—H3	119.2	C15—C16—H16	119.3
C3—C4—C5	119.9 (3)	C17—C16—H16	119.3
C3—C4—H4	120.0	C16—C17—C18	118.2 (3)
C5—C4—H4	120.0	C16—C17—C20	121.2 (3)
C6—C5—C4	119.0 (3)	C18—C17—C20	120.6 (3)
C6—C5—C8	118.2 (2)	C19—C18—C17	120.6 (3)
C4—C5—C8	122.6 (2)	C19—C18—H18	119.7
C7—C6—C5	120.3 (3)	C17—C18—H18	119.7
C7—C6—H6	119.9	C18—C19—C14	120.9 (3)
C5—C6—H6	119.9	C18—C19—H19	119.5
C6—C7—C2	121.6 (3)	C14—C19—H19	119.5
C6—C7—H7	119.2	C17—C20—H20A	109.5
C2—C7—H7	119.2	C17—C20—H20B	109.5
C2—C1—H1A	109.5	H20A—C20—H20B	109.5
C2—C1—H1B	109.5	C17—C20—H20C	109.5
H1A—C1—H1B	109.5	H20A—C20—H20C	109.5
C2—C1—H1C	109.5	H20B—C20—H20C	109.5
H1A—C1—H1C	109.5	N1—C26—C25	120.5 (4)
H1B—C1—H1C	109.5	N1—C26—H26	119.7
O1—C8—O2	122.8 (3)	C25—C26—H26	119.7
O1—C8—C5	125.1 (3)	C26—C25—C24	117.7 (4)
O2—C8—C5	112.1 (2)	C26—C25—H25	121.1
O2—C9—C11	111.0 (2)	C24—C25—H25	121.1
O2—C9—C10	106.4 (2)	C23—C24—C25	120.7 (4)
C11—C9—C10	114.1 (2)	C23—C24—H24	119.6
O2—C9—H9	108.4	C25—C24—H24	119.6
C11—C9—H9	108.4	C24—C23—C22	120.0 (4)
C10—C9—H9	108.4	C24—C23—H23	120.0
O3—C10—O4	125.2 (3)	C22—C23—H23	120.0
O3—C10—C9	121.6 (3)	N1—C22—C23	117.3 (3)
O4—C10—C9	113.0 (2)	N1—C22—C21	118.5 (3)
O7—C11—C9	107.58 (18)	C23—C22—C21	124.2 (3)
O7—C11—C12	112.05 (19)	C22—C21—H21A	109.5
C9—C11—C12	110.6 (2)	C22—C21—H21B	109.5
O7—C11—H11	108.8	H21A—C21—H21B	109.5
C9—C11—H11	108.8	C22—C21—H21C	109.5
C12—C11—H11	108.8	H21A—C21—H21C	109.5
O6—C12—O5	127.5 (2)	H21B—C21—H21C	109.5
O6—C12—C11	118.1 (2)	C22—N1—C26	123.6 (3)
O5—C12—C11	114.4 (2)	C22—N1—H1	122 (2)
O8—C13—O7	122.6 (2)	C26—N1—H1	114 (2)
O8—C13—C14	125.2 (3)	C8—O2—C9	118.4 (2)
O7—C13—C14	112.2 (2)	C10—O4—H4A	113 (3)
C15—C14—C19	118.7 (3)	C13—O7—C11	114.83 (19)
C15—C14—C13	123.1 (3)		
			
C7—C2—C3—C4	2.0 (5)	O8—C13—C14—C19	−7.5 (4)
C1—C2—C3—C4	−176.4 (3)	O7—C13—C14—C19	172.8 (2)
C2—C3—C4—C5	−0.1 (5)	C19—C14—C15—C16	1.4 (4)
C3—C4—C5—C6	−2.2 (4)	C13—C14—C15—C16	−179.5 (3)
C3—C4—C5—C8	173.3 (3)	C14—C15—C16—C17	−0.2 (5)
C4—C5—C6—C7	2.6 (5)	C15—C16—C17—C18	−0.4 (5)
C8—C5—C6—C7	−173.1 (3)	C15—C16—C17—C20	178.0 (3)
C5—C6—C7—C2	−0.7 (5)	C16—C17—C18—C19	−0.3 (5)
C3—C2—C7—C6	−1.6 (5)	C20—C17—C18—C19	−178.8 (3)
C1—C2—C7—C6	176.8 (3)	C17—C18—C19—C14	1.6 (5)
C6—C5—C8—O1	−3.3 (5)	C15—C14—C19—C18	−2.1 (5)
C4—C5—C8—O1	−178.8 (3)	C13—C14—C19—C18	178.8 (3)
C6—C5—C8—O2	175.6 (3)	N1—C26—C25—C24	−1.6 (6)
C4—C5—C8—O2	0.1 (4)	C26—C25—C24—C23	2.7 (6)
O2—C9—C10—O3	−31.1 (4)	C25—C24—C23—C22	−1.1 (6)
C11—C9—C10—O3	−153.9 (3)	C24—C23—C22—N1	−1.7 (5)
O2—C9—C10—O4	152.7 (2)	C24—C23—C22—C21	178.6 (4)
C11—C9—C10—O4	30.0 (3)	C23—C22—N1—C26	2.9 (4)
O2—C9—C11—O7	−56.2 (2)	C21—C22—N1—C26	−177.4 (3)
C10—C9—C11—O7	64.1 (3)	C25—C26—N1—C22	−1.2 (5)
O2—C9—C11—C12	66.5 (2)	O1—C8—O2—C9	12.3 (4)
C10—C9—C11—C12	−173.2 (2)	C5—C8—O2—C9	−166.7 (2)
O7—C11—C12—O6	19.8 (4)	C11—C9—O2—C8	−112.8 (2)
C9—C11—C12—O6	−100.2 (3)	C10—C9—O2—C8	122.5 (2)
O7—C11—C12—O5	−161.5 (2)	O8—C13—O7—C11	4.0 (4)
C9—C11—C12—O5	78.5 (3)	C14—C13—O7—C11	−176.33 (19)
O8—C13—C14—C15	173.4 (3)	C9—C11—O7—C13	−164.90 (19)
O7—C13—C14—C15	−6.2 (4)	C12—C11—O7—C13	73.3 (3)

### 2-Methylpyridinium hydrogen 2,3-bis(4-methylbenzoyloxy)succinate (I) Hydrogen-bond geometry (Å, º)

Cg1 is the centroid of the C2–C7 ring.

**Table:**

*D*—H···*A*	*D*—H	H···*A*	*D*···*A*	*D*—H···*A*
N1—H1···O5	0.87 (1)	1.74 (2)	2.593 (3)	166 (3)
O4—H4*A*···O6^i^	0.84 (1)	1.67 (1)	2.509 (2)	175 (5)
C1—H1*A*···O8^ii^	0.96	2.58	3.522 (4)	168
C16—H16···O1^ii^	0.93	2.51	3.362 (4)	153
C21—H21*A*···O3^iii^	0.96	2.38	3.238 (4)	148
C7—H7···*Cg*1^iv^	0.93	2.89	3.5882 (1)	133
C21—H21*B*···*Cg*1	0.96	2.91	3.7651 (1)	148

Symmetry codes: (i) *x*−1, *y*, *z*; (ii) −*x*+1, *y*−1/2, −*z*+1/2; (iii) −*x*+1, *y*+1/2, −*z*+1/2; (iv) −*x*, *y*+1/2, −*z*+1/2.

### Bis-[4-methylpyridinium hydrogen 2,3-bis(4-methylbenzoyloxy)succinate] pentahydrate (II) Crystal data

**Table:**

2C_20_H_17_O_8_^+^·2C_6_H_8_N^−^·5H_2_O	*Z* = 1
*M**_r_* = 1049.02	*F*(000) = 554
Triclinic, *P*1	*D*_x_ = 1.293 Mg m^−^^3^
*a* = 7.5106 (2) Å	Mo *K*α radiation, λ = 0.71073 Å
*b* = 10.0155 (3) Å	Cell parameters from 9138 reflections
*c* = 18.5203 (5) Å	θ = 2.3–23.5°
α = 75.646 (2)°	µ = 0.10 mm^−^^1^
β = 88.438 (2)°	*T* = 296 K
γ = 86.344 (2)°	Block, colourless
*V* = 1346.81 (7) Å^3^	0.40 × 0.30 × 0.30 mm

### Bis-[4-methylpyridinium hydrogen 2,3-bis(4-methylbenzoyloxy)succinate] pentahydrate (II) Data collection

**Table:**

Bruker APEXII CCD Diffractometer	6749 reflections with *I* > 2σ(*I*)
ω and φ scan	*R*_int_ = 0.031
Absorption correction: multi-scan (SADABS; Bruker, 2004)	θ_max_ = 25.0°, θ_min_ = 2.1°
*T*_min_ = 0.683, *T*_max_ = 0.746	*h* = −8→8
26169 measured reflections	*k* = −11→11
9433 independent reflections	*l* = −22→22

### Bis-[4-methylpyridinium hydrogen 2,3-bis(4-methylbenzoyloxy)succinate] pentahydrate (II) Refinement

**Table:**

Refinement on *F*^2^	H atoms treated by a mixture of independent and constrained refinement
Least-squares matrix: full	*w* = 1/[σ^2^(*F*_o_^2^) + (0.0673*P*)^2^] where *P* = (*F*_o_^2^ + 2*F*_c_^2^)/3
*R*[*F*^2^ > 2σ(*F*^2^)] = 0.044	(Δ/σ)_max_ < 0.001
*wR*(*F*^2^) = 0.121	Δρ_max_ = 0.37 e Å^−^^3^
*S* = 1.02	Δρ_min_ = −0.23 e Å^−^^3^
9433 reflections	Extinction correction: SHELXL-2016 (Sheldrick 2015), Fc^*^=kFc[1+0.001xFc^2^λ^3^/sin(2θ)]^-1/4^
715 parameters	Extinction coefficient: 0.032 (3)
21 restraints	Absolute structure: Flack *x* determined using 2690 quotients [(*I*^+^)-(*I*^-^)]/[(*I*^+^)+(*I*^-^)] (Parsons *et al.*, 2013)
Hydrogen site location: mixed	Absolute structure parameter: 0.6 (3)

### Bis-[4-methylpyridinium hydrogen 2,3-bis(4-methylbenzoyloxy)succinate] pentahydrate (II) Special details

**Table:**

Geometry. All esds (except the esd in the dihedral angle between two l.s. planes) are estimated using the full covariance matrix. The cell esds are taken into account individually in the estimation of esds in distances, angles and torsion angles; correlations between esds in cell parameters are only used when they are defined by crystal symmetry. An approximate (isotropic) treatment of cell esds is used for estimating esds involving l.s. planes.

### Bis-[4-methylpyridinium hydrogen 2,3-bis(4-methylbenzoyloxy)succinate] pentahydrate (II) Fractional atomic coordinates and isotropic or equivalent isotropic displacement parameters (Å^2^)

**Table:**

	*x*	*y*	*z*	*U*_iso_*/*U*_eq_	
C1	0.4416 (8)	0.4071 (7)	0.1428 (3)	0.0793 (17)	
H1A	0.515167	0.328752	0.168613	0.119*	
H1B	0.356832	0.377624	0.112988	0.119*	
H1C	0.515142	0.473133	0.111264	0.119*	
C2	0.3443 (7)	0.4722 (5)	0.1985 (3)	0.0532 (12)	
C3	0.3625 (7)	0.4182 (5)	0.2745 (3)	0.0554 (12)	
H3	0.440179	0.341349	0.291386	0.067*	
C4	0.2694 (6)	0.4746 (5)	0.3259 (2)	0.0480 (11)	
H4	0.283325	0.435386	0.376672	0.058*	
C5	0.1548 (6)	0.5900 (5)	0.3016 (2)	0.0402 (10)	
C6	0.1376 (6)	0.6468 (5)	0.2255 (2)	0.0501 (12)	
H6	0.063406	0.725793	0.208445	0.060*	
C7	0.2297 (7)	0.5868 (6)	0.1754 (2)	0.0565 (13)	
H7	0.214208	0.624703	0.124642	0.068*	
C8	0.0476 (6)	0.6537 (5)	0.3533 (2)	0.0412 (10)	
C9	−0.0219 (5)	0.6496 (4)	0.4789 (2)	0.0380 (10)	
H9	−0.028026	0.750688	0.462954	0.046*	
C10	−0.2101 (6)	0.5998 (5)	0.4849 (2)	0.0429 (10)	
C11	0.0718 (5)	0.6040 (4)	0.5525 (2)	0.0364 (9)	
H11	0.002250	0.640766	0.589553	0.044*	
C12	0.2588 (6)	0.6587 (5)	0.5467 (2)	0.0414 (10)	
C13	0.0855 (6)	0.3976 (5)	0.6475 (2)	0.0468 (11)	
C14	0.0943 (6)	0.2458 (5)	0.6654 (2)	0.0475 (11)	
C15	0.0686 (7)	0.1740 (5)	0.6117 (3)	0.0595 (13)	
H15	0.045114	0.222215	0.562792	0.071*	
C16	0.0777 (8)	0.0317 (6)	0.6302 (3)	0.0692 (15)	
H16	0.059400	−0.014355	0.593458	0.083*	
C17	0.1130 (8)	−0.0438 (5)	0.7013 (3)	0.0620 (13)	
C18	0.1433 (8)	0.0282 (6)	0.7543 (3)	0.0716 (16)	
H18	0.170779	−0.020632	0.802689	0.086*	
C19	0.1337 (8)	0.1714 (5)	0.7370 (3)	0.0645 (14)	
H19	0.153843	0.217304	0.773700	0.077*	
C20	0.1240 (10)	−0.1987 (6)	0.7208 (4)	0.0864 (19)	
H20A	0.149643	−0.233111	0.772822	0.130*	
H20B	0.012272	−0.230895	0.710246	0.130*	
H20C	0.217198	−0.231055	0.691735	0.130*	
C21	0.6306 (11)	−0.3317 (6)	0.7232 (4)	0.098 (2)	
H21A	0.668596	−0.372504	0.773301	0.147*	
H21B	0.516077	−0.363451	0.715858	0.147*	
H21C	0.715704	−0.357957	0.688824	0.147*	
C22	0.6171 (8)	−0.1767 (5)	0.7099 (3)	0.0630 (14)	
C23	0.5652 (8)	−0.0962 (6)	0.6422 (3)	0.0689 (15)	
H23	0.537456	−0.136144	0.604000	0.083*	
C24	0.5544 (8)	0.0457 (7)	0.6313 (3)	0.0759 (16)	
H24	0.520043	0.101361	0.585143	0.091*	
C25	0.6426 (9)	0.0252 (7)	0.7530 (4)	0.0848 (18)	
H25	0.668938	0.066964	0.790724	0.102*	
C26	0.6538 (8)	−0.1122 (6)	0.7648 (3)	0.0705 (15)	
H26	0.687538	−0.165771	0.811487	0.085*	
C27	0.6262 (8)	−0.4418 (6)	0.9500 (3)	0.0718 (15)	
H27A	0.725863	−0.399720	0.964770	0.108*	
H27B	0.613947	−0.531117	0.983246	0.108*	
H27C	0.645700	−0.451745	0.900093	0.108*	
C28	0.4590 (7)	−0.3526 (5)	0.9529 (3)	0.0509 (11)	
C29	0.2947 (7)	−0.3880 (5)	0.9345 (3)	0.0604 (13)	
H29	0.286017	−0.470171	0.920370	0.072*	
C30	0.1436 (7)	−0.3039 (5)	0.9365 (3)	0.0556 (12)	
H30	0.034756	−0.328088	0.922353	0.067*	
C31	0.1530 (6)	−0.1832 (4)	0.9597 (2)	0.0405 (10)	
C32	0.3143 (6)	−0.1487 (5)	0.9793 (3)	0.0485 (11)	
H32	0.321834	−0.068776	0.995697	0.058*	
C33	0.4662 (6)	−0.2312 (5)	0.9749 (3)	0.0537 (12)	
H33	0.575636	−0.204505	0.986997	0.064*	
C34	−0.0084 (6)	−0.0889 (4)	0.9560 (2)	0.0412 (10)	
C35	−0.1137 (5)	0.1186 (4)	0.9861 (2)	0.0373 (9)	
H35	−0.125725	0.153199	0.931976	0.045*	
C36	−0.2969 (6)	0.0908 (5)	1.0201 (2)	0.0422 (10)	
C37	−0.0266 (5)	0.2267 (4)	1.0163 (2)	0.0375 (9)	
H37	−0.086094	0.317571	0.995542	0.045*	
C38	0.1718 (6)	0.2300 (4)	0.9941 (3)	0.0408 (10)	
C39	−0.0725 (6)	0.2939 (5)	1.1298 (2)	0.0449 (11)	
C40	−0.1118 (5)	0.2436 (4)	1.2100 (2)	0.0406 (10)	
C41	−0.1176 (7)	0.3368 (5)	1.2554 (3)	0.0563 (13)	
H41	−0.094498	0.428711	1.234799	0.068*	
C42	−0.1569 (7)	0.2937 (6)	1.3295 (3)	0.0594 (13)	
H42	−0.159716	0.357109	1.358724	0.071*	
C43	−0.1928 (7)	0.1577 (6)	1.3625 (3)	0.0580 (13)	
C44	−0.1860 (7)	0.0669 (5)	1.3175 (3)	0.0578 (13)	
H44	−0.208865	−0.024948	1.338371	0.069*	
C45	−0.1464 (6)	0.1078 (5)	1.2426 (3)	0.0496 (11)	
H45	−0.142827	0.043780	1.213706	0.059*	
C46	−0.2300 (10)	0.1089 (7)	1.4447 (3)	0.0859 (19)	
H46A	−0.229866	0.185543	1.467232	0.129*	
H46B	−0.344544	0.069799	1.452475	0.129*	
H46C	−0.139480	0.040154	1.467038	0.129*	
C47	−0.7287 (12)	0.0319 (10)	1.3715 (6)	0.136 (3)	
H47A	−0.790137	0.114247	1.342928	0.204*	
H47B	−0.812131	−0.023849	1.403818	0.204*	
H47C	−0.638576	0.056479	1.400892	0.204*	
C48	−0.6451 (8)	−0.0458 (8)	1.3212 (4)	0.0847 (19)	
C49	−0.5482 (9)	−0.1688 (8)	1.3487 (4)	0.087 (2)	
H49	−0.540336	−0.203547	1.400063	0.104*	
C50	−0.4674 (10)	−0.2378 (9)	1.3051 (5)	0.095 (2)	
H50	−0.403703	−0.320975	1.325029	0.114*	
C51	−0.5745 (12)	−0.0702 (12)	1.1991 (5)	0.115 (3)	
H51	−0.581512	−0.038941	1.147539	0.139*	
C52	−0.6604 (9)	−0.0004 (9)	1.2439 (5)	0.105 (3)	
H52	−0.730884	0.078955	1.223287	0.126*	
N1	0.5918 (7)	0.1026 (5)	0.6848 (3)	0.0730 (13)	
N2	−0.4772 (8)	−0.1875 (8)	1.2309 (4)	0.1036 (19)	
O1	−0.0614 (5)	0.7494 (4)	0.33454 (18)	0.0651 (10)	
O2	0.0829 (4)	0.5948 (3)	0.42562 (14)	0.0419 (7)	
O3	−0.2586 (4)	0.5169 (4)	0.45325 (19)	0.0651 (10)	
O4	−0.3076 (4)	0.6597 (4)	0.52791 (19)	0.0626 (9)	
H4A	−0.402028	0.621747	0.537393	0.094*	
O5	0.2675 (4)	0.7843 (4)	0.5287 (2)	0.0632 (9)	
O6	0.3893 (4)	0.5698 (4)	0.55959 (18)	0.0569 (8)	
O7	0.0901 (6)	0.4658 (4)	0.69297 (17)	0.0749 (11)	
O8	0.0755 (4)	0.4557 (3)	0.57457 (14)	0.0423 (7)	
O9	−0.1347 (4)	−0.0835 (4)	0.91686 (18)	0.0609 (9)	
O10	0.0060 (3)	−0.0014 (3)	1.00073 (15)	0.0415 (7)	
O11	−0.3342 (4)	−0.0201 (3)	1.05921 (19)	0.0578 (8)	
O12	−0.4032 (4)	0.1993 (4)	1.0006 (2)	0.0639 (9)	
H12	−0.478807	0.199445	1.033417	0.096*	
O13	0.2042 (4)	0.2724 (4)	0.92676 (18)	0.0569 (8)	
O14	0.2833 (4)	0.1877 (3)	1.04482 (18)	0.0512 (8)	
O15	−0.0519 (4)	0.1899 (3)	1.09612 (14)	0.0409 (7)	
O16	−0.0613 (5)	0.4136 (4)	1.09713 (19)	0.0693 (10)	
H2	−0.402 (7)	−0.241 (5)	1.204 (3)	0.106 (17)*	
O17	−0.2550 (9)	−0.3002 (6)	1.1321 (3)	0.1186 (17)	
H17A	−0.199 (12)	−0.363 (6)	1.112 (4)	0.142*	
H17B	−0.263 (12)	−0.225 (5)	1.094 (3)	0.142*	
O18	−0.0808 (7)	0.4142 (7)	0.8384 (2)	0.123 (2)	
H18B	−0.036 (10)	0.453 (9)	0.796 (2)	0.147*	
H18A	0.000 (8)	0.383 (9)	0.869 (3)	0.147*	
O19	0.5407 (11)	0.3153 (8)	0.8300 (3)	0.145 (2)	
H19A	0.660 (5)	0.323 (12)	0.832 (3)	0.174*	
H19B	0.518 (9)	0.271 (10)	0.876 (3)	0.174*	
O20	0.5618 (8)	0.3695 (4)	0.6789 (3)	0.1005 (16)	
H20E	0.513 (10)	0.448 (5)	0.648 (3)	0.121*	
H20D	0.545 (10)	0.393 (7)	0.7215 (19)	0.121*	
O21	0.4508 (6)	0.3247 (4)	0.5022 (2)	0.0807 (12)	
H21E	0.554 (5)	0.355 (6)	0.492 (4)	0.097*	
H21D	0.383 (6)	0.375 (6)	0.520 (4)	0.097*	
H1	0.572 (7)	0.191 (3)	0.683 (3)	0.070 (17)*	

### Bis-[4-methylpyridinium hydrogen 2,3-bis(4-methylbenzoyloxy)succinate] pentahydrate (II) Atomic displacement parameters (Å^2^)

**Table:**

	*U*^11^	*U*^22^	*U*^33^	*U*^12^	*U*^13^	*U*^23^
C1	0.070 (4)	0.110 (5)	0.068 (3)	−0.006 (4)	0.014 (3)	−0.043 (3)
C2	0.047 (3)	0.067 (3)	0.050 (3)	−0.010 (3)	0.007 (2)	−0.022 (2)
C3	0.053 (3)	0.061 (3)	0.054 (3)	0.004 (3)	0.004 (2)	−0.019 (2)
C4	0.050 (3)	0.052 (3)	0.040 (2)	0.005 (2)	0.000 (2)	−0.009 (2)
C5	0.035 (2)	0.051 (3)	0.036 (2)	−0.004 (2)	−0.0002 (18)	−0.0120 (19)
C6	0.046 (3)	0.056 (3)	0.042 (3)	−0.003 (2)	−0.001 (2)	0.000 (2)
C7	0.056 (3)	0.079 (4)	0.032 (2)	−0.013 (3)	0.004 (2)	−0.009 (2)
C8	0.035 (2)	0.044 (3)	0.042 (2)	−0.002 (2)	−0.003 (2)	−0.007 (2)
C9	0.029 (2)	0.043 (2)	0.043 (2)	0.0018 (19)	0.0050 (18)	−0.0137 (19)
C10	0.035 (2)	0.052 (3)	0.041 (2)	−0.001 (2)	−0.0019 (19)	−0.010 (2)
C11	0.032 (2)	0.039 (2)	0.039 (2)	−0.0035 (19)	0.0043 (17)	−0.0111 (18)
C12	0.031 (2)	0.054 (3)	0.040 (2)	−0.004 (2)	−0.0010 (18)	−0.013 (2)
C13	0.049 (3)	0.051 (3)	0.039 (3)	−0.009 (2)	0.005 (2)	−0.009 (2)
C14	0.046 (3)	0.051 (3)	0.043 (2)	−0.008 (2)	0.006 (2)	−0.005 (2)
C15	0.075 (4)	0.048 (3)	0.055 (3)	−0.011 (3)	−0.011 (2)	−0.009 (2)
C16	0.079 (4)	0.064 (4)	0.069 (4)	−0.016 (3)	−0.013 (3)	−0.021 (3)
C17	0.065 (3)	0.047 (3)	0.072 (4)	−0.008 (3)	0.009 (3)	−0.009 (3)
C18	0.094 (4)	0.059 (4)	0.051 (3)	0.006 (3)	0.014 (3)	0.002 (3)
C19	0.085 (4)	0.063 (4)	0.044 (3)	−0.006 (3)	0.010 (3)	−0.010 (2)
C20	0.102 (5)	0.055 (3)	0.097 (5)	−0.007 (3)	0.011 (4)	−0.009 (3)
C21	0.136 (6)	0.052 (4)	0.104 (5)	−0.003 (4)	−0.013 (4)	−0.016 (3)
C22	0.071 (4)	0.049 (3)	0.067 (3)	−0.006 (3)	0.001 (3)	−0.011 (3)
C23	0.082 (4)	0.064 (4)	0.058 (3)	−0.006 (3)	−0.006 (3)	−0.010 (3)
C24	0.071 (4)	0.079 (4)	0.068 (4)	0.000 (3)	0.002 (3)	0.000 (3)
C25	0.096 (5)	0.077 (5)	0.090 (5)	−0.005 (4)	−0.016 (4)	−0.035 (4)
C26	0.087 (4)	0.065 (4)	0.059 (3)	0.000 (3)	−0.016 (3)	−0.015 (3)
C27	0.068 (4)	0.061 (3)	0.089 (4)	0.016 (3)	0.009 (3)	−0.027 (3)
C28	0.050 (3)	0.043 (3)	0.060 (3)	0.005 (2)	0.007 (2)	−0.015 (2)
C29	0.065 (3)	0.045 (3)	0.080 (3)	−0.003 (3)	0.005 (3)	−0.033 (3)
C30	0.048 (3)	0.053 (3)	0.073 (3)	−0.009 (2)	0.000 (2)	−0.027 (3)
C31	0.036 (2)	0.045 (2)	0.042 (2)	−0.005 (2)	0.0029 (18)	−0.0124 (19)
C32	0.041 (3)	0.042 (3)	0.067 (3)	0.005 (2)	−0.003 (2)	−0.024 (2)
C33	0.038 (3)	0.052 (3)	0.077 (3)	0.000 (2)	0.001 (2)	−0.030 (2)
C34	0.033 (2)	0.049 (3)	0.044 (2)	−0.002 (2)	0.001 (2)	−0.016 (2)
C35	0.025 (2)	0.048 (2)	0.038 (2)	0.0000 (19)	−0.0012 (16)	−0.0098 (18)
C36	0.028 (2)	0.050 (3)	0.049 (2)	−0.001 (2)	−0.001 (2)	−0.015 (2)
C37	0.027 (2)	0.044 (2)	0.038 (2)	0.0034 (18)	0.0004 (17)	−0.0060 (19)
C38	0.031 (2)	0.042 (2)	0.051 (3)	−0.006 (2)	0.008 (2)	−0.014 (2)
C39	0.038 (3)	0.049 (3)	0.052 (3)	−0.007 (2)	−0.003 (2)	−0.019 (2)
C40	0.032 (2)	0.048 (3)	0.044 (2)	−0.003 (2)	−0.0004 (18)	−0.015 (2)
C41	0.060 (3)	0.056 (3)	0.060 (3)	−0.009 (3)	0.001 (2)	−0.026 (2)
C42	0.067 (3)	0.064 (3)	0.057 (3)	−0.004 (3)	0.005 (2)	−0.034 (3)
C43	0.053 (3)	0.074 (4)	0.049 (3)	0.002 (3)	0.003 (2)	−0.021 (3)
C44	0.061 (3)	0.053 (3)	0.056 (3)	−0.003 (3)	0.002 (2)	−0.009 (2)
C45	0.053 (3)	0.048 (3)	0.052 (3)	−0.003 (2)	0.001 (2)	−0.020 (2)
C46	0.102 (5)	0.100 (5)	0.054 (3)	−0.005 (4)	0.015 (3)	−0.019 (3)
C47	0.092 (6)	0.129 (7)	0.165 (9)	−0.001 (5)	0.005 (6)	0.002 (7)
C48	0.047 (3)	0.106 (5)	0.087 (5)	−0.017 (4)	0.010 (3)	0.003 (4)
C49	0.055 (4)	0.095 (5)	0.091 (5)	−0.021 (4)	−0.016 (4)	0.019 (4)
C50	0.062 (4)	0.108 (6)	0.104 (6)	−0.014 (4)	−0.020 (4)	0.002 (5)
C51	0.074 (5)	0.156 (8)	0.088 (5)	−0.022 (6)	0.009 (5)	0.027 (6)
C52	0.055 (4)	0.122 (6)	0.101 (6)	−0.008 (4)	0.003 (4)	0.040 (5)
N1	0.077 (3)	0.047 (3)	0.096 (4)	−0.008 (3)	0.013 (3)	−0.018 (3)
N2	0.060 (4)	0.130 (6)	0.116 (6)	−0.032 (4)	0.005 (4)	−0.014 (5)
O1	0.071 (2)	0.066 (2)	0.0521 (19)	0.023 (2)	−0.0061 (17)	−0.0089 (16)
O2	0.0363 (16)	0.0536 (18)	0.0340 (15)	0.0039 (14)	0.0027 (12)	−0.0094 (13)
O3	0.051 (2)	0.081 (2)	0.076 (2)	−0.0193 (19)	0.0088 (17)	−0.042 (2)
O4	0.0320 (17)	0.089 (3)	0.080 (2)	−0.0067 (17)	0.0121 (16)	−0.045 (2)
O5	0.048 (2)	0.057 (2)	0.087 (2)	−0.0149 (17)	−0.0003 (18)	−0.0182 (18)
O6	0.0296 (17)	0.072 (2)	0.066 (2)	−0.0004 (17)	0.0003 (14)	−0.0113 (17)
O7	0.128 (3)	0.058 (2)	0.0402 (18)	−0.007 (2)	0.0018 (19)	−0.0143 (16)
O8	0.0449 (17)	0.0438 (17)	0.0377 (16)	−0.0053 (14)	0.0003 (13)	−0.0089 (13)
O9	0.045 (2)	0.081 (2)	0.065 (2)	0.0000 (17)	−0.0126 (17)	−0.0335 (18)
O10	0.0332 (16)	0.0478 (18)	0.0450 (16)	0.0081 (14)	−0.0070 (13)	−0.0164 (13)
O11	0.0451 (19)	0.055 (2)	0.067 (2)	−0.0086 (16)	0.0124 (16)	−0.0047 (18)
O12	0.0275 (17)	0.062 (2)	0.095 (3)	0.0001 (16)	0.0108 (16)	−0.0071 (19)
O13	0.0412 (19)	0.072 (2)	0.054 (2)	−0.0065 (16)	0.0116 (15)	−0.0092 (16)
O14	0.0242 (15)	0.072 (2)	0.0581 (18)	0.0020 (15)	−0.0043 (14)	−0.0187 (16)
O15	0.0349 (16)	0.0486 (17)	0.0404 (15)	0.0000 (13)	0.0038 (12)	−0.0141 (14)
O16	0.098 (3)	0.052 (2)	0.060 (2)	−0.020 (2)	0.008 (2)	−0.0156 (18)
O17	0.119 (5)	0.115 (4)	0.106 (4)	0.004 (4)	−0.017 (3)	0.002 (3)
O18	0.122 (4)	0.168 (5)	0.054 (2)	0.073 (4)	0.009 (3)	−0.006 (3)
O19	0.223 (8)	0.147 (5)	0.069 (3)	−0.015 (6)	−0.010 (4)	−0.031 (3)
O20	0.154 (5)	0.066 (3)	0.084 (3)	0.006 (3)	−0.025 (3)	−0.026 (2)
O21	0.089 (3)	0.067 (3)	0.084 (3)	−0.005 (2)	0.012 (2)	−0.016 (2)

### Bis-[4-methylpyridinium hydrogen 2,3-bis(4-methylbenzoyloxy)succinate] pentahydrate (II) Geometric parameters (Å, º)

**Table:**

C1—C2	1.502 (7)	C29—H29	0.9300
C1—H1A	0.9600	C30—C31	1.385 (6)
C1—H1B	0.9600	C30—H30	0.9300
C1—H1C	0.9600	C31—C32	1.363 (6)
C2—C7	1.376 (7)	C31—C34	1.481 (6)
C2—C3	1.384 (7)	C32—C33	1.378 (6)
C3—C4	1.377 (6)	C32—H32	0.9300
C3—H3	0.9300	C33—H33	0.9300
C4—C5	1.384 (6)	C34—O9	1.201 (5)
C4—H4	0.9300	C34—O10	1.357 (5)
C5—C6	1.389 (6)	C35—O10	1.428 (5)
C5—C8	1.473 (6)	C35—C36	1.511 (6)
C6—C7	1.375 (7)	C35—C37	1.525 (6)
C6—H6	0.9300	C35—H35	0.9800
C7—H7	0.9300	C36—O11	1.211 (5)
C8—O1	1.208 (5)	C36—O12	1.288 (6)
C8—O2	1.349 (5)	C37—O15	1.442 (5)
C9—O2	1.437 (5)	C37—C38	1.536 (6)
C9—C11	1.505 (6)	C37—H37	0.9800
C9—C10	1.520 (6)	C38—O13	1.236 (5)
C9—H9	0.9800	C38—O14	1.250 (5)
C10—O3	1.207 (5)	C39—O16	1.209 (5)
C10—O4	1.296 (5)	C39—O15	1.339 (5)
C11—O8	1.439 (5)	C39—C40	1.474 (6)
C11—C12	1.531 (6)	C40—C45	1.382 (6)
C11—H11	0.9800	C40—C41	1.401 (6)
C12—O5	1.224 (5)	C41—C42	1.363 (7)
C12—O6	1.267 (5)	C41—H41	0.9300
C13—O7	1.211 (5)	C42—C43	1.388 (8)
C13—O8	1.334 (5)	C42—H42	0.9300
C13—C14	1.471 (6)	C43—C44	1.375 (7)
C14—C19	1.381 (7)	C43—C46	1.505 (7)
C14—C15	1.388 (7)	C44—C45	1.376 (7)
C15—C16	1.378 (7)	C44—H44	0.9300
C15—H15	0.9300	C45—H45	0.9300
C16—C17	1.370 (7)	C46—H46A	0.9600
C16—H16	0.9300	C46—H46B	0.9600
C17—C18	1.385 (8)	C46—H46C	0.9600
C17—C20	1.500 (8)	C47—C48	1.461 (12)
C18—C19	1.387 (7)	C47—H47A	0.9600
C18—H18	0.9300	C47—H47B	0.9600
C19—H19	0.9300	C47—H47C	0.9600
C20—H20A	0.9600	C48—C49	1.379 (10)
C20—H20B	0.9600	C48—C52	1.396 (10)
C20—H20C	0.9600	C49—C50	1.303 (11)
C21—C22	1.508 (8)	C49—H49	0.9300
C21—H21A	0.9600	C50—N2	1.344 (10)
C21—H21B	0.9600	C50—H50	0.9300
C21—H21C	0.9600	C51—C52	1.339 (12)
C22—C23	1.363 (7)	C51—N2	1.353 (11)
C22—C26	1.375 (7)	C51—H51	0.9300
C23—C24	1.382 (8)	C52—H52	0.9300
C23—H23	0.9300	N1—H1	0.88 (3)
C24—N1	1.303 (8)	N2—H2	0.97 (3)
C24—H24	0.9300	O4—H4A	0.8200
C25—C26	1.336 (8)	O12—H12	0.8200
C25—N1	1.359 (8)	O17—H17A	0.88 (3)
C25—H25	0.9300	O17—H17B	0.89 (3)
C26—H26	0.9300	O18—H18B	0.85 (3)
C27—C28	1.502 (7)	O18—H18A	0.84 (3)
C27—H27A	0.9600	O19—H19A	0.91 (3)
C27—H27B	0.9600	O19—H19B	0.88 (3)
C27—H27C	0.9600	O20—H20E	0.91 (3)
C28—C29	1.380 (7)	O20—H20D	0.88 (3)
C28—C33	1.379 (6)	O21—H21E	0.85 (3)
C29—C30	1.375 (7)	O21—H21D	0.82 (3)
			
C2—C1—H1A	109.5	C30—C29—C28	121.2 (4)
C2—C1—H1B	109.5	C30—C29—H29	119.4
H1A—C1—H1B	109.5	C28—C29—H29	119.4
C2—C1—H1C	109.5	C29—C30—C31	120.2 (4)
H1A—C1—H1C	109.5	C29—C30—H30	119.9
H1B—C1—H1C	109.5	C31—C30—H30	119.9
C7—C2—C3	117.4 (4)	C32—C31—C30	118.9 (4)
C7—C2—C1	120.8 (5)	C32—C31—C34	121.5 (4)
C3—C2—C1	121.8 (5)	C30—C31—C34	119.3 (4)
C4—C3—C2	122.2 (5)	C31—C32—C33	120.6 (4)
C4—C3—H3	118.9	C31—C32—H32	119.7
C2—C3—H3	118.9	C33—C32—H32	119.7
C3—C4—C5	119.5 (4)	C32—C33—C28	121.2 (4)
C3—C4—H4	120.2	C32—C33—H33	119.4
C5—C4—H4	120.2	C28—C33—H33	119.4
C4—C5—C6	119.0 (4)	O9—C34—O10	122.6 (4)
C4—C5—C8	122.5 (4)	O9—C34—C31	125.9 (4)
C6—C5—C8	118.5 (4)	O10—C34—C31	111.4 (3)
C7—C6—C5	120.2 (5)	O10—C35—C36	113.0 (4)
C7—C6—H6	119.9	O10—C35—C37	106.7 (3)
C5—C6—H6	119.9	C36—C35—C37	111.2 (3)
C6—C7—C2	121.6 (4)	O10—C35—H35	108.6
C6—C7—H7	119.2	C36—C35—H35	108.6
C2—C7—H7	119.2	C37—C35—H35	108.6
O1—C8—O2	122.0 (4)	O11—C36—O12	126.4 (4)
O1—C8—C5	124.8 (4)	O11—C36—C35	123.0 (4)
O2—C8—C5	113.2 (4)	O12—C36—C35	110.5 (4)
O2—C9—C11	107.5 (3)	O15—C37—C35	107.1 (3)
O2—C9—C10	110.8 (3)	O15—C37—C38	111.9 (3)
C11—C9—C10	111.2 (3)	C35—C37—C38	109.8 (3)
O2—C9—H9	109.1	O15—C37—H37	109.3
C11—C9—H9	109.1	C35—C37—H37	109.3
C10—C9—H9	109.1	C38—C37—H37	109.3
O3—C10—O4	126.0 (4)	O13—C38—O14	126.7 (4)
O3—C10—C9	123.8 (4)	O13—C38—C37	115.7 (4)
O4—C10—C9	110.2 (4)	O14—C38—C37	117.5 (4)
O8—C11—C9	107.4 (3)	O16—C39—O15	123.2 (4)
O8—C11—C12	112.4 (3)	O16—C39—C40	125.2 (4)
C9—C11—C12	110.9 (3)	O15—C39—C40	111.6 (4)
O8—C11—H11	108.7	C45—C40—C41	118.2 (4)
C9—C11—H11	108.7	C45—C40—C39	122.7 (4)
C12—C11—H11	108.7	C41—C40—C39	119.1 (4)
O5—C12—O6	126.4 (4)	C42—C41—C40	120.4 (5)
O5—C12—C11	116.6 (4)	C42—C41—H41	119.8
O6—C12—C11	117.0 (4)	C40—C41—H41	119.8
O7—C13—O8	121.9 (4)	C41—C42—C43	121.7 (4)
O7—C13—C14	124.8 (4)	C41—C42—H42	119.1
O8—C13—C14	113.2 (4)	C43—C42—H42	119.1
C19—C14—C15	118.4 (4)	C44—C43—C42	117.4 (4)
C19—C14—C13	119.6 (4)	C44—C43—C46	120.8 (5)
C15—C14—C13	121.9 (4)	C42—C43—C46	121.8 (5)
C16—C15—C14	120.5 (5)	C45—C44—C43	122.0 (5)
C16—C15—H15	119.8	C45—C44—H44	119.0
C14—C15—H15	119.8	C43—C44—H44	119.0
C17—C16—C15	121.9 (5)	C44—C45—C40	120.3 (4)
C17—C16—H16	119.1	C44—C45—H45	119.8
C15—C16—H16	119.1	C40—C45—H45	119.8
C16—C17—C18	117.4 (5)	C43—C46—H46A	109.5
C16—C17—C20	121.4 (5)	C43—C46—H46B	109.5
C18—C17—C20	121.1 (5)	H46A—C46—H46B	109.5
C17—C18—C19	121.7 (5)	C43—C46—H46C	109.5
C17—C18—H18	119.1	H46A—C46—H46C	109.5
C19—C18—H18	119.1	H46B—C46—H46C	109.5
C14—C19—C18	120.0 (5)	C48—C47—H47A	109.5
C14—C19—H19	120.0	C48—C47—H47B	109.5
C18—C19—H19	120.0	H47A—C47—H47B	109.5
C17—C20—H20A	109.5	C48—C47—H47C	109.5
C17—C20—H20B	109.5	H47A—C47—H47C	109.5
H20A—C20—H20B	109.5	H47B—C47—H47C	109.5
C17—C20—H20C	109.5	C49—C48—C52	117.1 (8)
H20A—C20—H20C	109.5	C49—C48—C47	120.8 (7)
H20B—C20—H20C	109.5	C52—C48—C47	122.1 (8)
C22—C21—H21A	109.5	C50—C49—C48	122.0 (7)
C22—C21—H21B	109.5	C50—C49—H49	119.0
H21A—C21—H21B	109.5	C48—C49—H49	119.0
C22—C21—H21C	109.5	C49—C50—N2	119.2 (8)
H21A—C21—H21C	109.5	C49—C50—H50	120.4
H21B—C21—H21C	109.5	N2—C50—H50	120.4
C23—C22—C26	118.0 (5)	C52—C51—N2	118.2 (8)
C23—C22—C21	120.1 (5)	C52—C51—H51	120.9
C26—C22—C21	121.9 (5)	N2—C51—H51	120.9
C22—C23—C24	119.1 (5)	C51—C52—C48	120.6 (8)
C22—C23—H23	120.5	C51—C52—H52	119.7
C24—C23—H23	120.5	C48—C52—H52	119.7
N1—C24—C23	120.9 (5)	C24—N1—C25	121.5 (5)
N1—C24—H24	119.6	C24—N1—H1	124 (4)
C23—C24—H24	119.6	C25—N1—H1	114 (4)
C26—C25—N1	118.6 (5)	C50—N2—C51	122.6 (8)
C26—C25—H25	120.7	C50—N2—H2	112 (3)
N1—C25—H25	120.7	C51—N2—H2	125 (3)
C25—C26—C22	122.0 (5)	C8—O2—C9	115.8 (3)
C25—C26—H26	119.0	C10—O4—H4A	109.5
C22—C26—H26	119.0	C13—O8—C11	116.5 (3)
C28—C27—H27A	109.5	C34—O10—C35	116.2 (3)
C28—C27—H27B	109.5	C36—O12—H12	109.5
H27A—C27—H27B	109.5	C39—O15—C37	116.9 (3)
C28—C27—H27C	109.5	H17A—O17—H17B	104 (4)
H27A—C27—H27C	109.5	H18B—O18—H18A	110 (5)
H27B—C27—H27C	109.5	H19A—O19—H19B	100 (4)
C29—C28—C33	117.8 (4)	H20E—O20—H20D	98 (4)
C29—C28—C27	122.1 (4)	H21E—O21—H21D	114 (5)
C33—C28—C27	120.1 (5)		
			
C7—C2—C3—C4	0.7 (7)	C32—C31—C34—O9	152.2 (5)
C1—C2—C3—C4	−177.8 (5)	C30—C31—C34—O9	−22.1 (7)
C2—C3—C4—C5	−0.8 (8)	C32—C31—C34—O10	−24.7 (6)
C3—C4—C5—C6	−0.4 (7)	C30—C31—C34—O10	160.9 (4)
C3—C4—C5—C8	178.4 (4)	O10—C35—C36—O11	3.9 (5)
C4—C5—C6—C7	1.7 (7)	C37—C35—C36—O11	−116.0 (4)
C8—C5—C6—C7	−177.3 (4)	O10—C35—C36—O12	−175.1 (3)
C5—C6—C7—C2	−1.7 (7)	C37—C35—C36—O12	64.9 (4)
C3—C2—C7—C6	0.6 (7)	O10—C35—C37—O15	−77.4 (4)
C1—C2—C7—C6	179.1 (5)	C36—C35—C37—O15	46.2 (4)
C4—C5—C8—O1	−175.5 (4)	O10—C35—C37—C38	44.3 (4)
C6—C5—C8—O1	3.4 (7)	C36—C35—C37—C38	167.9 (4)
C4—C5—C8—O2	4.7 (6)	O15—C37—C38—O13	−173.9 (3)
C6—C5—C8—O2	−176.4 (4)	C35—C37—C38—O13	67.3 (5)
O2—C9—C10—O3	5.4 (6)	O15—C37—C38—O14	7.4 (5)
C11—C9—C10—O3	−114.2 (5)	C35—C37—C38—O14	−111.4 (4)
O2—C9—C10—O4	−174.2 (3)	O16—C39—C40—C45	171.2 (5)
C11—C9—C10—O4	66.2 (4)	O15—C39—C40—C45	−8.2 (6)
O2—C9—C11—O8	−62.8 (4)	O16—C39—C40—C41	−7.7 (7)
C10—C9—C11—O8	58.7 (4)	O15—C39—C40—C41	172.9 (4)
O2—C9—C11—C12	60.4 (4)	C45—C40—C41—C42	−0.2 (7)
C10—C9—C11—C12	−178.1 (4)	C39—C40—C41—C42	178.8 (5)
O8—C11—C12—O5	−179.4 (3)	C40—C41—C42—C43	−0.2 (8)
C9—C11—C12—O5	60.3 (5)	C41—C42—C43—C44	0.5 (8)
O8—C11—C12—O6	1.7 (5)	C41—C42—C43—C46	177.9 (5)
C9—C11—C12—O6	−118.5 (4)	C42—C43—C44—C45	−0.4 (8)
O7—C13—C14—C19	−9.0 (8)	C46—C43—C44—C45	−177.8 (5)
O8—C13—C14—C19	169.6 (4)	C43—C44—C45—C40	0.0 (8)
O7—C13—C14—C15	172.7 (5)	C41—C40—C45—C44	0.3 (7)
O8—C13—C14—C15	−8.6 (6)	C39—C40—C45—C44	−178.6 (4)
C19—C14—C15—C16	1.7 (8)	C52—C48—C49—C50	2.8 (10)
C13—C14—C15—C16	180.0 (5)	C47—C48—C49—C50	−177.7 (7)
C14—C15—C16—C17	−0.4 (9)	C48—C49—C50—N2	0.5 (10)
C15—C16—C17—C18	−1.3 (9)	N2—C51—C52—C48	1.9 (12)
C15—C16—C17—C20	−179.5 (6)	C49—C48—C52—C51	−4.1 (10)
C16—C17—C18—C19	1.7 (9)	C47—C48—C52—C51	176.5 (8)
C20—C17—C18—C19	179.9 (6)	C23—C24—N1—C25	0.0 (9)
C15—C14—C19—C18	−1.3 (8)	C26—C25—N1—C24	0.0 (10)
C13—C14—C19—C18	−179.6 (5)	C49—C50—N2—C51	−3.0 (10)
C17—C18—C19—C14	−0.4 (9)	C52—C51—N2—C50	1.7 (11)
C26—C22—C23—C24	1.0 (9)	O1—C8—O2—C9	2.2 (6)
C21—C22—C23—C24	−179.8 (6)	C5—C8—O2—C9	−178.0 (3)
C22—C23—C24—N1	−0.5 (9)	C11—C9—O2—C8	−162.9 (3)
N1—C25—C26—C22	0.5 (10)	C10—C9—O2—C8	75.3 (4)
C23—C22—C26—C25	−1.0 (9)	O7—C13—O8—C11	0.5 (6)
C21—C22—C26—C25	179.7 (7)	C14—C13—O8—C11	−178.2 (3)
C33—C28—C29—C30	1.2 (8)	C9—C11—O8—C13	−154.2 (3)
C27—C28—C29—C30	−179.1 (5)	C12—C11—O8—C13	83.6 (4)
C28—C29—C30—C31	−2.1 (8)	O9—C34—O10—C35	−14.8 (6)
C29—C30—C31—C32	0.9 (7)	C31—C34—O10—C35	162.2 (3)
C29—C30—C31—C34	175.4 (4)	C36—C35—O10—C34	81.4 (4)
C30—C31—C32—C33	1.1 (7)	C37—C35—O10—C34	−156.1 (3)
C34—C31—C32—C33	−173.2 (4)	O16—C39—O15—C37	−4.1 (6)
C31—C32—C33—C28	−2.0 (8)	C40—C39—O15—C37	175.3 (3)
C29—C28—C33—C32	0.8 (7)	C35—C37—O15—C39	−146.9 (3)
C27—C28—C33—C32	−178.9 (5)	C38—C37—O15—C39	92.8 (4)

### Bis-[4-methylpyridinium hydrogen 2,3-bis(4-methylbenzoyloxy)succinate] pentahydrate (II) Hydrogen-bond geometry (Å, º)

Cg1 and Cg4 are the centroids of the C2–C7 and C40–C45 rings, respectively.

**Table:**

*D*—H···*A*	*D*—H	H···*A*	*D*···*A*	*D*—H···*A*
C24—H24···O21	0.93	2.41	3.262 (8)	152
C51—H51···O11	0.93	2.43	3.072 (10)	127
O4—H4*A*···O6^i^	0.82	1.69	2.503 (4)	170
O12—H12···O14^i^	0.82	1.80	2.472 (4)	138
N2—H2···O17	0.97 (3)	1.89 (3)	2.832 (10)	164 (5)
O17—H17*A*···O16^ii^	0.88 (3)	2.48 (3)	3.330 (7)	162 (7)
O17—H17*B*···O11	0.89 (3)	2.03 (4)	2.828 (7)	148 (7)
O18—H18*B*···O7	0.85 (3)	2.09 (5)	2.894 (6)	157 (9)
O18—H18*A*···O13	0.84 (3)	2.00 (4)	2.815 (6)	164 (9)
O19—H19*A*···O18^iii^	0.91 (3)	2.22 (5)	3.090 (11)	160 (11)
O19—H19*B*···O12^iii^	0.88 (3)	2.32 (5)	3.115 (7)	151 (7)
O19—H19*B*···O13	0.88 (3)	2.52 (6)	3.048 (8)	120 (6)
O20—H20*E*···O6	0.91 (3)	2.00 (3)	2.867 (6)	159 (6)
O20—H20*D*···O19	0.88 (3)	1.97 (4)	2.717 (7)	142 (6)
O21—H21*E*···O3^iii^	0.85 (3)	2.19 (4)	2.976 (5)	154 (6)
O21—H21*D*···O6	0.82 (3)	2.25 (5)	2.914 (5)	139 (5)
N1—H1···O20	0.88 (3)	1.77 (3)	2.644 (6)	172 (6)
C41—H41···*Cg*1^iv^	0.93	2.90	3.468 (6)	121
C47—H47*A*···*Cg*4^i^	0.96	2.94	3.707 (10)	137

Symmetry codes: (i) *x*−1, *y*, *z*; (ii) *x*, *y*−1, *z*; (iii) *x*+1, *y*, *z*; (iv) *x*, *y*, *z*+1.
